# Recurrent diabetic ketoacidosis

**DOI:** 10.20945/2359-3997000000158

**Published:** 2019-07-11

**Authors:** Evgenia Brandstaetter, Carmi Bartal, Iftach Sagy, Alan Jotkowitz, Leonid Barski

**Affiliations:** 1 Department of Internal Medicine F Soroka University Medical Center Beer-Sheva Israel Department of Internal Medicine F, Soroka University Medical Center, Beer-Sheva, Israel; 2 Department of Internal Medicine E Soroka University Medical Center Beer-Sheva Israel Department of Internal Medicine E, Soroka University Medical Center, Beer-Sheva, Israel; 3 Clinical Research Center Soroka University Medical Center Beer Sheva Israel Clinical Research Center, Soroka University Medical Center, Beer Sheva, Israel

**Keywords:** Recurrent DKA, high-risk patients, readmission DKA patients

## Abstract

**Objective:**

The purpose of this study is to examine risk factors for recurrence of diabetic ketoacidosis and determine interventions to prevent future admissions.

**Materials and methods:**

Review article.

**Results:**

Recurrent diabetic ketoacidosis is a serious and not uncommon health problem. Diabetic ketoacidosis is associated with severe morbidity and mortality and hospital admissions due to this problem constitute a serious economic burden on the healthcare system. Younger age at diabetic ketoacidosis onset, poor baseline glycemic control and elevated HbA1C, patient comorbidities, depression, alcohol or substance abuse, particularly active cocaine use, have been associated with recurrent diabetic ketoacidosis. In addition, socioeconomic factors (such as ethnic minority status, use of public health insurance and underinsurance), psychosocial, economic, and behavioral factors (including financial constraint, stretching a limited insulin supply, and homelessness) have been all reported to be associated with readmission among diabetic ketoacidosis patients.

**Conclusions:**

Identifying high-risk patients during the first diabetic ketoacidosis admission and performing relevant interventions (repeated instructions of insulin use, social help and involvement of family members in medical treatment, collaboration with the patient’s primary care physician in order to establish a close and frequent follow up program) may help prevent future admissions. Further studies need to take place to determine whether early interventions with those factors prevent future admissions.

## INTRODUCTION

Diabetic ketoacidosis (DKA) is a life-threatening complication of diabetes. It mainly occurs in patients with type 1 diabetes, but it is not uncommon in some patients with type 2 diabetes.

DKA is a state of absolute or relative insulin deficiency potentiated by glucose counter-regulatory hormone excess. The most common causes are inadequate insulin treatment, medical illness and new onset of diabetes. DKA consists of the triad of hyperglycemia, ketonemia, and an anion gap metabolic acidosis. The severity of DKA is classified as mild, moderate or severe based on the intensity of the metabolic acidosis and the presence of altered mental status (1).

DKA is associated with severe morbidity and mortality. Furthermore, hospital admissions due to this problem constitute a serious economic burden on the healthcare system. According to The Center for Disease Control and Prevention (CDC) – National Diabetes Surveillance Program, hospital discharges with DKA as the first-listed diagnosis increased from about 80,000 discharges in 1988 to about 140,000 in 2009 (2). Mortality rate of DKA has fallen significantly in the last 20 years, from 7.96% to 0.67%, as reported by Lin and cols. (3); although in developing countries it is still high (4). Treatment of DKA is expensive, accounting for an estimated total cost of 2.4 billion dollars annually in the US (5). Analysis of recurrent admissions has the potential to help identify factors that could assist physicians and hospitals in obviating readmission for DKA (6).

## RISK FACTORS AND CAUSES OF RECURRENT DKA ADMISSION

Among the known risk factors for DKA some may allegedly assist physicians to prevent re-admissions (6). The common risk factors of recurrent DKA are shown in Table 1.

**Table 1:** Risk factors for recurrent DKA

Patient characteristics
Young age
Male sex
Patient comorbidities
Psychiatric illness (i.e. depression)
Alcohol or substance abuse (i.e. active cocaine use)
Chronic medical illness (i.e. hypertension, dyslipidemia, coronary artery disease)
Socioeconomic factors
Ethnic minority status
Use of public health insurance
Underinsurance
Financial constraint
Stretching a limited insulin supply
Homelessness
Other
Noncompliance with medical treatment
Changing pen devices for insulin injection
Incorrect injection technique of the insulin

**Diabetes characteristics**

Poor baseline glycemic control
Elevated glycated hemoglobin (HbA1C)

For example, younger age at DKA onset has been associated with increased risk of DKA recurrence (7,8). Other studies note that readmitted DKA patients are more often male than female (9), while a rising trend in DKA readmission among female diabetic patients less than 35 years of age has also been noted (5). Studies also suggest that poor baseline glycemic control and elevated glycated hemoglobin (HbA1C) have been associated with recurrent DKA (7,10). Among patient comorbidities, depression, alcohol or substance abuse seem to play important roles (7), particularly active cocaine use which was strongly associated with DKA readmission (11,12). In addition, socioeconomic factors (such as ethnic minority status, use of public health insurance and underinsurance), psychosocial, economic, and behavioral factors (including financial constraint, stretching a limited insulin supply, and homelessness) have been all reported to be associated with readmission among DKA patients (6,7,8,10,13).

**Figure 1:**
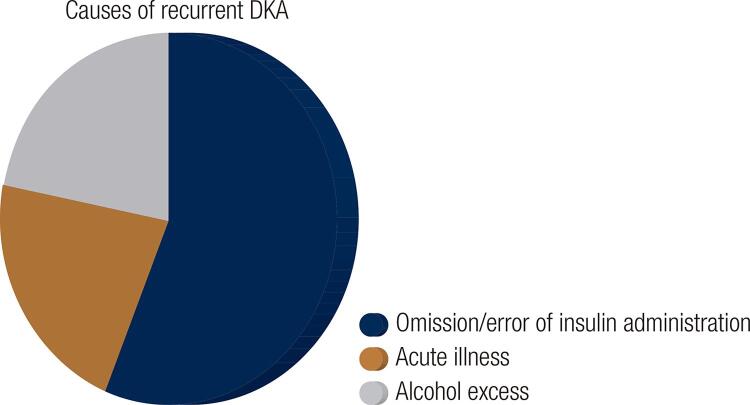
Causes of recurrent DKA

Several studies have examined the causes for recurrent DKA admission. We performed a literature review seeking information regarding those risk factors and their possible prevention/management. All the studies were retrospective case-control/chart-review studies, although case-reports were also noted. The common causes of recurrent DKA are shown in Figure 1.

Bradford AL and cols. conducted a retrospective case-control study via chart review at a tertiary academic medical center. Six potential risk factors for DKA readmission (age < 35 years, history of depression, HbA1C > 10.6% on admission, substance or alcohol abuse history, ethnic minority status, and self-pay or publicly funded insurance) were examined and scored dichotomously. Each individual risk factor, as well as the total score of significant individual risk factors, was compared for those patients readmitted for DKA during the 5-year study period vs. those who were not readmitted during the same period.

The study included 355 patients diagnosed with DKA. Twenty seven percent of the study group was readmitted for DKA (or HSS) during the study period. It was found that there were significantly increased odds of readmission for DKA with four out of six risk factors: age < 35 years (OR: 3.021, 95% CI: 1.878-4.866), history of depression (OR: 3.465, 95% CI: 2.085-5.758), substance or alcohol abuse history (OR: 2.828, 95% CI: 1.736-4.606) and self-pay or publicly funded insurance (OR: 1.773, 95% CI: 1.051-2.99). Using the variables found to exert a significant effect on readmission odds, a total “ABCD” score (A: age, B: behavioral health, C: coverage, D: drug or alcohol abuse) was calculated. This ABCD score was found to have a significant effect on odds of readmission (OR: 2.508, 95% CI: 1.918-3.280) (6).

Lohiya and cols. also conducted a retrospective chart review study of all patients with admissions for recurrent DKA (one or more previous episodes) in two private community teaching hospitals in Birmingham, Alabama, during a 6-year study period. Demographic, medical and insurance data were obtained from the medical records.

The study included 80 patients with recurrent DKA. Sixty one percent were African American and 64% were women. Seventy nine percent had received prior diabetes education, and 76% were insured. Eighty three percent of the patients had comorbidities, such as hypertension, dyslipidemia, coronary artery disease and chronic kidney disease. The average HbA1C was 11.1%. The most common psychiatric disorder was depression (31%). Alcohol and/or substance abuse was present in 24.5% of patients. Forty percent had a combination of the precipitating factors for DKA. Omission of insulin was the leading cause of recurrent DKA (Figure 1); 44% reported omission of insulin because of illness and 40% gave no reason for stopping their insulin. Twenty percent reported financial difficulty in obtaining their medications. Five percent reported insulin pump malfunction (14).

Skinner (15) discusses the possible reasons for insulin omission among young people, who are at increased risk for recurrent DKA. First, eating disorders or weight manipulations in adolescent girls is an important consideration (16). Second, omission of treatment may result from the desire or need to escape the parental home (perhaps associated with sexual, physical or emotional abuse or neglect). Third, there may also be depression, which is 2-3 times more prevalent in people with diabetes (17,18). And finally, the very process of learning to live with and cope with a life-limiting condition can lead to feelings of resentment and denial, and periods of rebellion against diabetes.

Randall and cols. interviewed patients to understand behavioral, socioeconomic, and psychosocial factors of recurrent DKA. Patients were interviewed to collect information on demographics, duration of diabetes, medical treatment, known diabetes complications, history of receiving diabetes education, and precipitating factors. Medical records were also reviewed. Out of 164 subjects 91 had recurrent DKA admissions at the time of enrolment (first admission with DKA was reported in 73 patients). Insulin discontinuation was the most common major precipitating cause in both patients with first-time and recurrent episodes of DKA (56% and 78%, respectively; 68% overall). Of the patients who discontinued insulin, 34% “just stopped” (giving no clear reason for stopping), 26% lacked money to buy insulin, 17% felt too sick to take the insulin, 15% stated that their insulin supply was lost or stolen or they were away from it, and 8% were lowering their dose to stretch their supply. The next most common cause was medical illness (18% of those with first-time DKA and 20% of those with recurrent DKA). Most patients (62%) were uninsured, only 17% had stable employment and lack of social support was common. Psychiatric illness was prevalent (46% of patients had a history of depression) and significantly more patients with recurrent DKA had abused drugs compared with those with first time DKA. In addition, compared with first-time DKA, patients with recurrent DKA episodes were more likely to be homeless or have experienced homelessness. Patient characteristics were further analyzed by the number of DKA admissions at the index admission, in categories of 0, 1-4, 5-10, and > 10. Patients with multiple episodes were leaner, had longer duration of diabetes, and developed diabetes at a younger age. They also had a higher rate of having received diabetes education; however, there was no significant increase in knowledge of the meaning of A1C as the number of admissions increased. Moreover, homelessness and drug abuse were associated with increased DKA re-admissions (5).

Cooper H and cols. demonstrated similar results in an observational retrospective case-control study conducted in Auckland, New Zealand (80 patients – 40 with more than one admission with DKA within 5 years and 40 with a single admission during that time): alcohol or substance abuse, history of psychiatric illness and psychotropic medication use were present in 42.5% of patients readmitted due to DKA (in comparison with only 35% of the patients with one admission with DKA over the 5-year study period). The identified precipitant for ketoacidosis was most commonly omission/error of insulin administration, followed by acute illness and alcohol excess (19).

Two specific risk factors for recurrent DKA were proposed by Nyenwe and cols. and Bhardwaj and cols.: active use of cocaine and changing pen devices for insulin injection, respectively (20,21).

Nyenwe and cols. performed a retrospective analysis of sequential adult admissions for DKA at Bronx Lebanon Hospital Center in New York between 2001 and 2004. They concluded that active use of cocaine is an independent risk factor for recurrent DKA, as are noncompliance and Hispanic ethnicity. Of these 3 factors, cocaine showed the strongest association with DKA (20).

Bhardwaj and cols. present two case reports of a 68-year-old man and a 17-year-old boy with recurrent episodes of DKA due to incorrect injection technique of the insulin via FlexPen (21).

Sodium-glucose cotransporter 2 inhibitors (SGLT2i) are a new class of oral hypoglycemic drugs recently introduced into clinical practice for the management of type 2 DM (22-24). These drugs prevent the reabsorption of glucose at the proximal renal tubules by targeting SGLT2 (22-25). Their favorable clinical profile has led to an increased interest in SGLT2i by health care providers and the wide use of these agents in clinical practice. SGLT2i have been associated with euglycemic diabetic ketoacidosis, a life-threatening emergency characterized by hyperglycemia with blood glucose levels < 200 mg/dL. This can result in delayed diagnosis and treatment with the potential for adverse metabolic consequences (25,26). We did not find in the literature cases of recurrent DKA among patients treated by SGLT2i, but the use of these drugs may lead to the development of potentially dangerous complications including DKA. Patients taking SGLT2i who become ill should discontinue the medication, undergo prompt ketone evaluation and start basal insulin, if ketones are positive. In addition, patients should be educated to stop their SGLT2 inhibitor at least 1 week prior to elective procedures (26).

In conclusion, recurrent DKA is a serious and not uncommon health problem. Several risk factors for recurrence have been identified. Some of those are not easily controlled (chronic disease, psychiatric illness, socioeconomic status) but others are modifiable: omission/error of insulin administration, compliance with treatment, drug or alcohol abuse, insurance status.

Identifying high-risk patients during the first DKA admission and performing relevant interventions (such as repeated instructions of insulin use, social help, involvement of family members in medical treatment, collaboration with the patient’s primary care physician in order to establish a close and frequent follow up program) may help prevent future admissions.

In the studies performed to date there was no homogeneity regarding the patients’ disease (type 1 or type 2 diabetes mellitus) and the time frame defining recurrence. Furthermore, only one study distinguished between patients with different recurrence rates, and no study performed a prospective follow up of patients after a recurrence-preventing intervention.

Further studies need to take place to determine whether early interventions with those factors prevent future admissions.
